# Growth, Properties and Applications of Bi_0.5_Na_0.5_TiO_3_ Ferroelectric Nanomaterials

**DOI:** 10.3390/nano11071724

**Published:** 2021-06-30

**Authors:** Yuan Liu, Yun Ji, Ya Yang

**Affiliations:** 1 Center on Nanoenergy Research, School of Physical Science and Technology, Guangxi University, Nanning 530004, China; liuyuan1@binn.cas.cn; 2 CAS Center for Excellence in Nanoscience, Beijing Key Laboratory of Micro-Nano Energy and Sensor, Beijing Institute of Nanoenergy and Nanosystems, Chinese Academy of Sciences, Beijing 100083, China; jiyun@binn.cas.cn; 3 School of Nanoscience and Technology, University of Chinese Academy of Sciences, Beijing 100049, China

**Keywords:** BNT nanomaterials, ferroelectricity, piezoelectricity, pyroelectricity, dielectric property

## Abstract

The emerging demands for miniaturization of electronics has driven the research into various nanomaterials. Lead-free Bi_0.5_Na_0.5_TiO_3_ (BNT) ferroelectric nanomaterials have drawn great interest owing to their superiorities of large remanent polarization, high pyroelectric and piezoelectric coefficients, unique photovoltaic performance and excellent dielectric properties. As attractive multifunctional ferroelectrics, BNT nanomaterials are widely utilized in various fields, such as energy harvest, energy storage, catalysis as well as sensing. The growing desire for precisely controlling the properties of BNT nanomaterials has led to significant advancements in material design and preparation approaches. BNT ferroelectric nanomaterials exhibit significant potential in fabrication of electronic devices and degradation of waste water, which pushes forward the advancement of the Internet of things and sustainable human development. This article presents an overview of research progresses of BNT ferroelectric nanomaterials, including growth, properties and applications. In addition, future prospects are discussed.

## 1. Introduction

As fascinating multifunctional materials, ferroelectrics have been widely utilized in optoelectronic devices [1,2], capacitors [3], energy harvesters [4,5], oscillators [6], transducers [7] and sensors [8,9]. For a long time, Pb(Zr_x_Ti_1−x_)O_3_ (PZT) ferroelectric materials have been intensively investigated since they possess large piezoelectric coefficient together with high Curie temperature. However, containing Pb element, PZT materials may do harm to ambient environment and human health, which impedes their further development in daily applications. Investigations of high-performance Pb-free ferroelectric nanomaterials, such as BaTiO_3_ (BT) [10,11], Na-doped KNbO_3_ (KNN) [12], Bi_0.5_Na_0.5_TiO_3_ (BNT) [13,14] as well as BiFeO_3_ (BFO) [15], have become a vital topic. BNT ferroelectric nanomaterials have attracted considerable attention since they were first synthesized in the year of 1960 by Smolenskii et al. [16]. Owing to large remanent polarization, high Curie temperature, high pyroelectric and piezoelectric coefficients, unique photovoltaic performance and excellent dielectric properties, BNT ferroelectric nanomaterials are regarded as excellent substitutes for PZT materials. The properties of BNT ferroelectric nanomaterials exhibit a strong dependence upon their structure and morphology. In order to obtain specific functions, BNT ferroelectric nanomaterials have been devised into ceramics, films and nanostructures (such as nanoparticles and nanowires), and fabricated by various routes, such as solid-state sintering process, aqueous chemical solution deposition and electrospinning technology. In addition to structure and morphology, composition also plays an essential role in determination of the BNT ferroelectric nanomaterials’ intrinsic properties. For instance, as compared with pure BNT, Sn modified BNT ferroelectric nanomaterials possess more excellent dielectric properties [17], and Sr^2+^ ions substituted BNT ferroelectric nanomaterials show higher recoverable energy density *W*_rec_ as well as larger efficiency *η* [18]. Moreover, construction of solid solutions and composites materials provides another effective approach for the improvement of BNT ferroelectric nanomaterials’ properties [19,20]. As a vital member of lead-free ferroelectrics, BNT ferroelectric nanomaterials can not only be utilized in a variety of electronic devices, but also can work as catalyst to purify waste water, pushing forward the advancement of the Internet of things and sustainable human development.

This paper reviews BNT ferroelectric nanomaterials with a detailed look at their basic properties together with most recent progresses. The review begins with descriptions about material growth. And then, overviews of properties, including ferroelectricity, dielectricity, piezoelectricity, pyroelectricity and photovoltaic property are described. Subsequently, a variety of recent applications with focus on energy harvest, energy storage, catalysis and motion monitoring are introduced. In the end, the future development and challenges are discussed.

## 2. Growth

**Figure 1:**
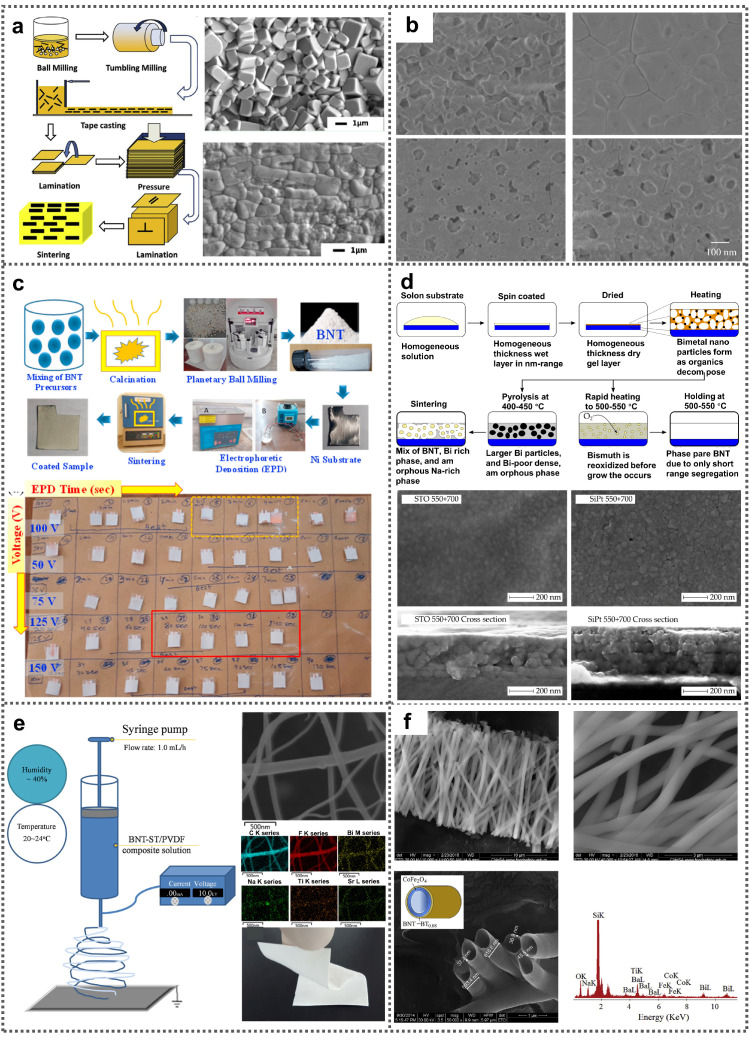
Material design and preparation of BNT ferroelectric nanomaterials. (**a**) Fabrication process and SEM images of <001> textured piezoelectric BNT-BKT-xST ceramic samples prepared through template-aided grain growth method (Reproduced with permission from [28], Elsevier, 2014). (**b**) SEM images of piezoelectric BNT thin films deposited via sol-gel technology at various temperature (Reproduced with permission from [35], Elsevier, 2016). (**c**) Preparation progress of BNT films by electrophoretic deposition technology and photographs of resulted films at different applied voltages (Reproduced with permission from [36], Elsevier, 2020). (**d**) Fabrication schematic of BNT films through chemical solution deposition method (Reproduced with permission from [37], MDPI, 2017). (**e**) Preparation progress of 0.78BNT-0.22ST nanofibers by electrospinning technology, and SEM images and photograph of flexible piezoelectric 0.78BNT-0.22ST/PVDF composite membrane (Reproduced with permission from [47], Elsevier, 2016). (**f**) SEM images of piezoelectric/ferromagnetic 0.92BNT-0.08BT/CoFe_2_O_4_ coaxial core-shell nanotubes (Reproduced with permission from [48], Elsevier, 2018).

Structure, morphology and composition act as vital factors to determine the properties of BNT ferroelectric nanomaterials. To achieve various functions, diverse BNT ferroelectric nanomaterials such as ceramics, thin films and nanostructures have been fabricated [21,22]. Nowadays, a variety of approaches, including solid-phase reaction method, hydrothermal method, reactive-templated grain growth method, aqueous chemical solution deposition, sol-gel method and electrospinning method, have been developed to prepare BNT ferroelectric nanomaterials. Solid-phase reaction method, sol-gel method and hydrothermal process are most commonly utilized approaches to obtain BNT powders. As compared with the other two methods, solid-phase reaction method is ease of operation, exhibiting superiority for large quantity production of BNT powders. Sol-gel method and hydrothermal process provide convenient ways for preparing high-purity BNT powders with smaller dimensions. In recent years, pure BNT ferroelectric ceramics have been intensively fabricated through a high-temperature sintering process using BNT powders as the starting materials. By pressing BNT powders into pellets and sintering the samples at about 1150 °C, compact BNT ceramics can be obtained. However, due to volatilization of Bi^3+^/Na^+^ and accumulation of oxygen vacancies during high-temperature sintering treatment, secondary phases are easily formed. To improve the stability of BNT ceramics, several effective approaches have been proposed, such as construction of A-/B-site ion substituted BNT ceramics, and fabrication of BNT-based solid solution by alloying with other ferroelectrics [23,24,25,26]. Mahmood et al. synthesized piezoelectric Bi_0.5_Na_0.5_TiO_3_-xBaTiO_3_ (BNT-xBT) ceramics which possessed a relatively high density (~96%) [27]. Morphtropic phase boundary (MPB) region where rhombohedral phase together with tetragonal phase can be simultaneously observed was obtained at x = 0.06 and 0.07. Bai et al. devised <001> textured (1 − x)(0.83Bi_0.5_Na_0.5_TiO_3_-0.17Bi_0.5_K_0.5_TiO_3_)-xSrTiO_3_ (BNT-BKT-xST) ceramic disks using plate-shaped ST as template (Figure 1a) [28]. Utilizing 9–15 mol% ST template, <001> oriented particles led to textured samples which had brick wall-like microstructure and extremely high texture degree (more than 90% Lotgering factor). BNT ceramics have the superiorities of ease of fabrication and low-cost, however, their large dimensions and high hardness are adverse to their applications for future miniaturized electronic devices [29,30,31,32,33,34]. BNT thin films possess nano/micron scale thicknesses, which are more suitable for fabrication of nano/microelectronics. Dargham et al. successfully prepared piezoelectric BNT thin film with rhombohedral perovskite phase (340 nm in thickness) by using Pt/TiO_2_/SiO_2_/Si as substrate through sol-gel technology [35]. By optimizing annealing temperature, the density and crystallinity of the BNT films were greatly improved (Figure 1b). Rafiq et al. prepared BNT film on a flexible Ni substrate by utilizing electrophoretic deposition technology [36]. The thickness and adhesion of the BNT film strongly depended on the applied voltage during the electrophoretic deposition process. By increasing the applied voltage to 125 V, thick (165 μm) and well-covered BNT films can be realized (Figure 1c). Christensen et al. prepared piezoelectric BNT thin films with perovskite phase on ST and Si/Pt substrates through chemical solution deposition method. Aqueous solution containing ethanolamine, sodium hydroxide, bismuth citrate, titanium tetraisopropoxide and citric acid was utilized as the precursor [37]. Figure 1d exhibits the detailed fabrication process of the BNT films. By modulating pyrolyzation temperature and sintering temperature to 550 °C and 700 °C, respectively, BNT films with excellent uniformity and high densification were obtained (Figure 1d). In addition to ceramics and thin films, BNT nanostructures including nanorods, nanoballs, nanowires and nanosheets have been devised and prepared [38,39,40,41,42,43,44,45,46]. For example, Ji et al. fabricated 0.78BNT-0.22ST nanofibers by electrospinning technology, and fabricated flexible piezoelectric composite membrane by embedding the 0.78BNT-0.22ST nanofibers into PVDF polymers, as shown in Figure 1e [47]. Moreover, piezoelectric/ferromagnetic 0.92BNT-0.08BT/CoFe_2_O_4_ coaxial core-shell nanotubes were successfully prepared with the help of polycarbonate membrane templates, as exhibited in Figure 1f [48].

In conclusion, different BNT nanomaterials prepared in different ways have differences in their morphology and other structures, which is an effective way to expand the application of BNT nanomaterials. We have summarized the preparation method, size and other relevant information of BNT nanomaterials with different morphologies in Table 1, so that everyone can see it directly.

## 3. Properties

Properties of BNT ferroelectric nanomaterials are mainly determined by structure, morphology, composition and ambient temperature. In this section, we review the ferroelectricity, piezoelectricity, dielectric property, pyroelectricity and photovoltaic property of BNT ferroelectric nanomaterials.

### 3.1. Ferroelectricity

**Figure 2:**
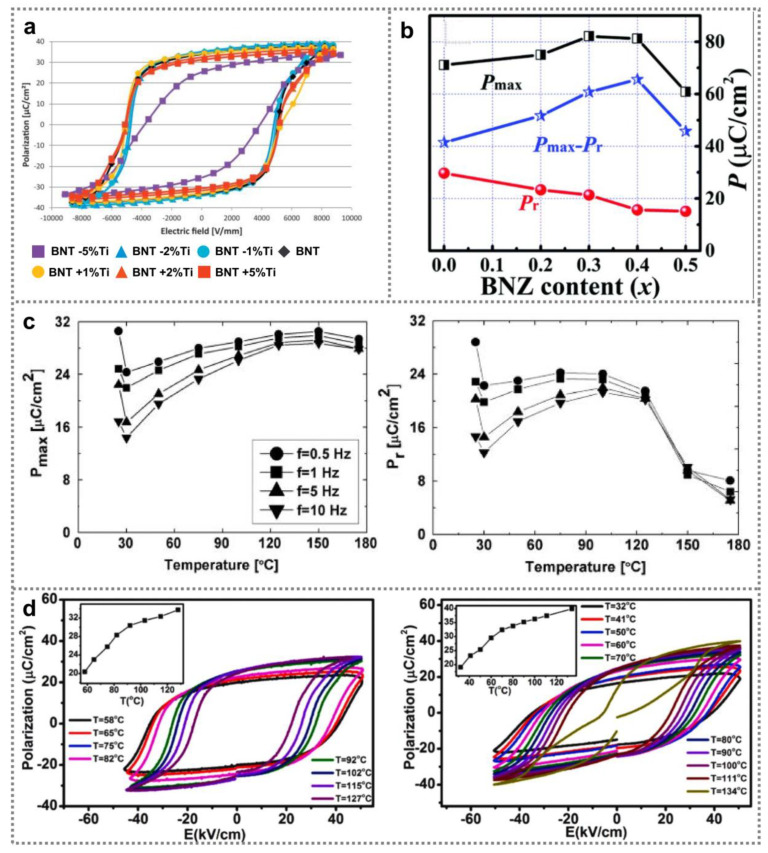
Ferroelectricity of BNT ferroelectric nanomaterials. (**a**) *P*-*E* hysteresis loop from BNT ceramic samples containing various amount of Ti (Reproduced with permission from [50], Elsevier, 2014). (**b**) *P*_max_, *P*_r_ and *P*_max_–*P*_r_ of BNT-xBNZ film under 2200 kV cm^–1^ electric field (Reproduced with permission from [52], RSC advances, 2018). (**c**) *P*_r_ of 0.95(0.94BNT-0.06BT)-0.05CaTiO_3_ ceramic samples as a function of temperature (Reproduced with copyright permission from [55], AIP Publishing, 2013) (**d**) BNT-xBT ceramic disks’ *P*-*E* loops at different temperature, and corresponding *P*_r_ of the samples as a function of temperature (Reproduced with permission from [56], Elsevier, 2019).

Polarization-electric field (*P*-*E*) hysteresis loops provide an effective way to directly characterize ferroelectricity of materials [49]. Naderer et al. studied the ferroelectricity of BNT ceramic samples which contain various amount of titanium utilizing *P*-*E* loops. As shown in Figure 2a, 1–2% Ti-deficiency led to lower coercive field and higher remanent polarization [50]. Moreover, Ti content also has a remarkable influence on the stability of electric field-induced ferroelectric state in BNT materials [51]. Sun et al. analyzed ferroelectricity of (1 − x)Bi_0.5_Na_0.5_TiO_3_-xBiNi_0.5_Zr_0.5_O_3_ films (BNT-xBNZ) [52]. Figure 2b depicts the dependence of relevant largest polarization *P*_max_ together with remnant polarization *P*_r_ of the BNT-xBNZ films on BNZ content. Obviously, with increasing BNZ content, *P*_max_ rises to its maximum (65.6 mC cm^−2^) at x of 0.4 and then declines with further increasing x, meanwhile, *P*_r_ gradually reduces. On one hand, compressive stress can be created by B-site substitution, which promotes the domain reversal under electric field, leading to promoted *P*_max_ with increasing x. On the other hand, B-site substitution can also result in local random electric field, which helps the polarization recover to the quondam state, consequently, *P*_r_ decreases at larger BNZ content [53]. It was reported that the remnant polarization of BNT nanomaterials showed a strong relevance with temperature [54]. As shown in Figure 2c, promotion of temperature contributes to higher maximum polarization *P*_max_ as well as higher remnant polarization *P*_r_ in 0.95(0.94BNT-0.06BT)-0.05CaTiO_3_ ceramics [55], which can be ascribed to heating-induced short-range ergodic relaxation phase transition of long-range ferroelectrics. Similar phenomena were also observed in Bi_0.5_(Na_1 − x_K_x_)_0.5_TiO_3_ ceramics (Figure 2e) [56].

All in all, BNT nanomaterials substituted by A and B sites or doped with other systems will have a certain impact on their ferroelectric properties such as saturated polarization P_max_, residual polarization P_r_, coercive field E_c_, energy storage density W_rec_, etc. It is an important factor to change the ferroelectric properties of BNT nanomaterials. Here, we summarize the relevant ferroelectric properties reported in Table 2. In summary, the modification or doping of BNT nanomaterials has the potential to change its ferroelectric properties.

### 3.2. Dielectric Property

**Figure 3:**
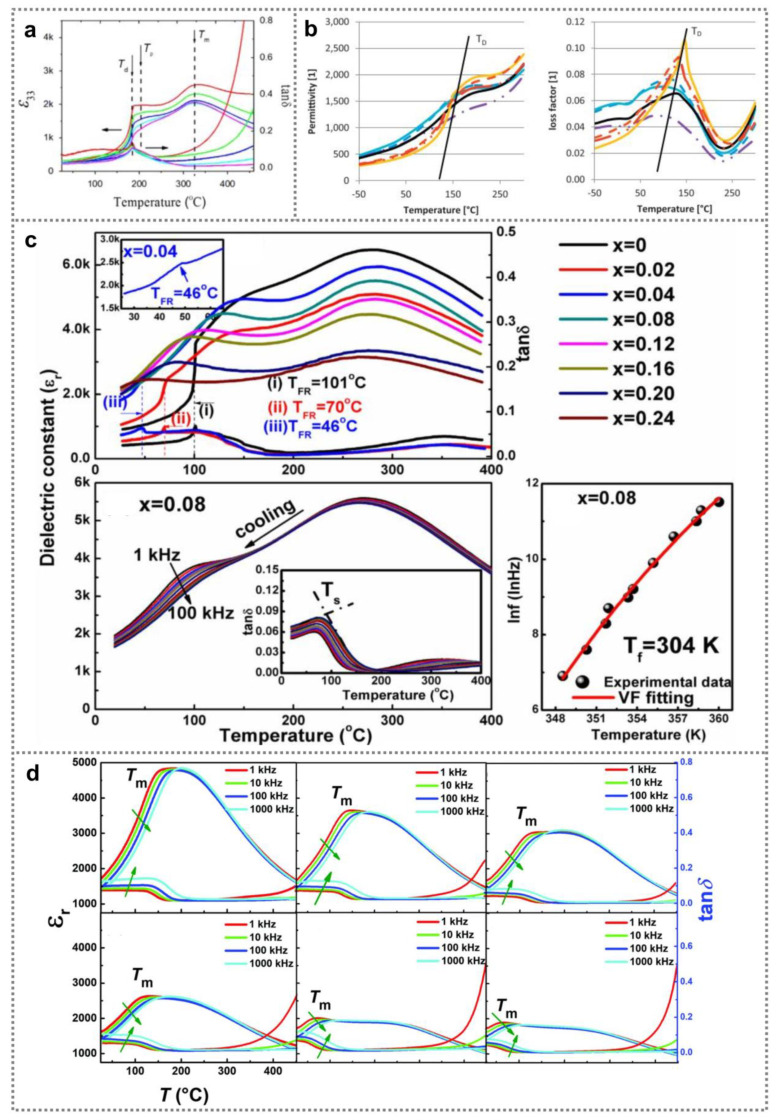
Dielectric property of BNT ferroelectric nanomaterials. (**a**) Typical dielectric constant and loss factor of BNT-based ceramic samples (Reproduced with permission from [58], Elsevier, 2014). (**b**) Dielectric constant and loss factor of BNT ceramic samples containing various amount of Ti (Reproduced with permission from [50], Elsevier, 2014). (**c**) Dielectric constant as well as dielectric loss of BNT-BT-xSBT ceramics containing various amount of SBT, and frequency-dependent dielectric property of BNT-BT-0.08SBT ceramic samples (Reproduced with permission from [54], Elsevier, 2017). (**d**) Dielectric properties of BNT-ST-100xAN ceramics with different AN content (Reproduced with permission from [23], RSC advances, 2018).

Generally, two main dielectric anomalies can be observed for BNT-based ferroelectric nanomaterials [57,60,61,62,63], as illustrated in Figure 3a [58]. The temperature *T*_d_ is called the depolarization temperature that is deduced according to the first peak of loss factor tan*δ.* BNT-based nanomaterials can change from ferroelectric state to relaxed state when the temperature is above *T*_d_. The second main anomalous dielectric peak at *T*_m_ is related to the transition to paraelectric phase. Temperature-related dielectric constant as well as loss factor of BNT ceramic samples containing various amount Ti have been investigated [50]. It was found that dielectric constant as well as loss factor of Ti-rich BNT samples increased much faster than that of Ti-deficient samples as temperature promoted. In addition, *T*_d_ can be increased by increasing Ti content, and obtain its maximum value of 143 °C in BNT ceramics with 5% Ti-excess (Figure 3b). Li et al. investigated SBT content-related dielectric property of BNT-BT-xSBT materials, as shown in Figure 3c [57]. It can be seen that addition of SBT can decrease the dielectric constant. Figure 3d shows the dielectric properties of (1 − x)(0.76Bi_0.5_Na_0.5_TiO_3_-0.24SrTiO_3_)-xAgNbO_3_ (BNT-ST-xAN) ceramic samples at various temperature (25–450 °C). The BNT-ST-xAN ceramic samples’ dielectric constant can maintain at large values over a wide temperature range because of locally coexisting polar nano-regions of different phases. With promoting the AN content, local random fields can be created due to random distribution of Ag^+^ ions, Nb^5+^ ions as well as vacancies, breaking the macroscopic long-range ferroelectric sequence. Consequently, the largest dielectric constant is promoted with increasing the AN content [23]. Additionally, polarization process can dislocate Ti ions from the B-sites of BNT-based materials, resulting in charged dislocation defects. Owing to the strong interaction between the charged dislocation defects and domain walls, the dielectric constants as well as dielectric loss can be improved [59,64].

### 3.3. Piezoelectricity

**Figure 4:**
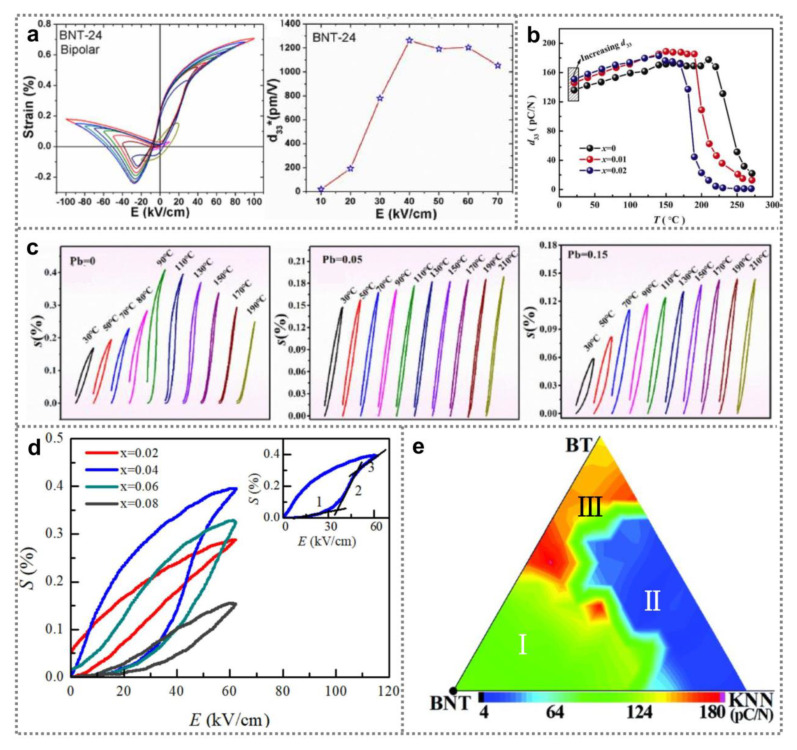
Piezoelectricity of BNT ferroelectric nanomaterials. (**a**) Bipolar *S*-*E* patterns of Sr_0.24_(Bi_0.76_Na_0.73_Li_0.03_)_0.5_TiO_3_ ceramics (Reproduced with permission from [65], Elsevier, 2020). (**b**) Piezoelectric coefficients *d*_33_ of 0.875(Bi_0.5_Na_0.5_)TiO_3_0.125BaTiO_3_-xKNbO_3_ nanomaterials (x = 0, 0.01 and 0.02) as a function of temperature (Reproduced with permission from [72], Elsevier, 2020). (**c**) Unipolar strain *S*-*E* curves of Pb-doped ceramic samples with various Pb content at different temperature (Reproduced with permission from [73], Elsevier, 2020). (**d**) Unipolar *S*-*E* curves of Bi_0.5_Na_0.5_TiO_3_-Bi_0.5_K_0.5_TiO_3_-xBi(Mg_0.75_Ta_0.25_)O_3_ ceramic samples (x = 0.02, 0.04, 0.06, 0.08) (Reproduced with permission from [74], Elsevier, 2020). (**e**) Piezoelectric coefficient of (100 − x − y)BNT-xBT-yKNN ceramics with different composition (Reproduced with permission from [75], RSC advances, 2020).

Piezoelectricity of BNT ferroelectric nanomaterials exhibits strong dependence on material composition, external electric fields and temperature [65,66,67,68]. Field-induced strain of ferroelectric materials reflects the deformation magnitude of the materials with applied electric field [22,69,70]. Figure 4a depicts bipolar strain-electric field patterns (*S*-*E*) from ferroelectric Sr_0.24_(Bi_0.76_Na_0.73_Li_0.03_)_0.5_TiO_3_ ceramics [65]. Because of strong internal bias fields from charged defects (oxygen vacancies), the *S*-*E* patterns show representative butterfly-like shape with negative strain, and exhibit asymmetry as the applied electric field changes [65,66,67]. Takenaka et al. improved the piezoelectricity of (Bi_0.5_Na_0.5_)TiO_3_-(Bi_0.5_K_0.5_)TiO_3_-BaTiO_3_ ceramic samples by constructing MPB composition, obtaining a large piezoelectric coefficient *d*_33_ (182 pC N^−1^) [71]. Wei et al. evaluated the thermal stability of the piezoelectricity in 0.875Bi_0.5_Na_0.5_TiO_3_-0.125BaTiO_3−_xKNbO_3_ nanomaterials [72]. As shown in Figure 4b, introducing KNbO_3_ can effectively promote the piezoelectric coefficient *d*_33_; however, lowing the depolarization temperature of the samples. By optimizing the x value to 0.01, the piezoelectric coefficient *d*_33_ of the samples was promoted from 135 pC N^−1^ to 147 pC N^−1^, maintaining at a large value (187 pC N^−1^) near depolarization temperature. Han et al. investigated the influence of Pb content on piezoelectricity of BNT-based nanomaterials [73]. Figure 4c depicts the *S*-*E* patterns of the samples with x of 0, 0.05 and 0.15 at various temperature. As temperature increases, a maximum unipolar strain can be obtained from Pb-free sample near the depolarization temperature (77 °C). However, the unipolar strain for the samples with x of 0.05 and 0.15 monotonically rises with rising the temperature, suggesting the depolarization temperature is increased due to addition of Pb. By optimizing x to 0.15, a large *d*_33_ (140 pC N^−1^) was achieved. Figure 4d depicts the *S*-*E* patterns of Bi_0.5_Na_0.5_TiO_3_-Bi_0.5_K_0.5_TiO_3_-xBi(Mg_0.75_Ta_0.25_)O_3_ ceramic samples [74]. With increasing x to 0.04, ergodic relaxor-ferroelectric phase transition occurs, resulting in typical hysteresis behavior of unipolar *S*-*E* curve, large unipolar strain value (0.4%) as well as large inverse piezoelectric coefficient (632 pm V^−1^). Figure 4e shows the impact of phase transition on (100 − x − y)BNT-xBT-yKNN ceramics’ piezoelectric coefficient [75], indicating that the maximum piezoelectric coefficient (181 pC N^−1^) can be obtained near the boundary between I region and II region, with corresponding to 91BNT-6BT-3KNN ceramics. In addition, it was reported that macroscopic polarization induced by strong external electric fields and magnetic domains also determined the piezoelectricity of BNT ferroelectric nanomaterials [76].

Here, we summarize the relevant piezoelectric and dielectric properties of BNT nanomaterials reported in Table 3. These include electrostriction coefficient Q_33_, electrostrain S, dielectric constant ε_r_, and piezoelectric constants d_33_ and d_33_*. What can be seen is the doped or modified BNT nanomaterials, and its related piezoelectric and dielectric properties will change accordingly. Some excellent properties are also being used in fields such as piezoelectric sensors or frequency sensors.

### 3.4. Pyroelectric and Photovoltaic Properties

**Figure 5:**
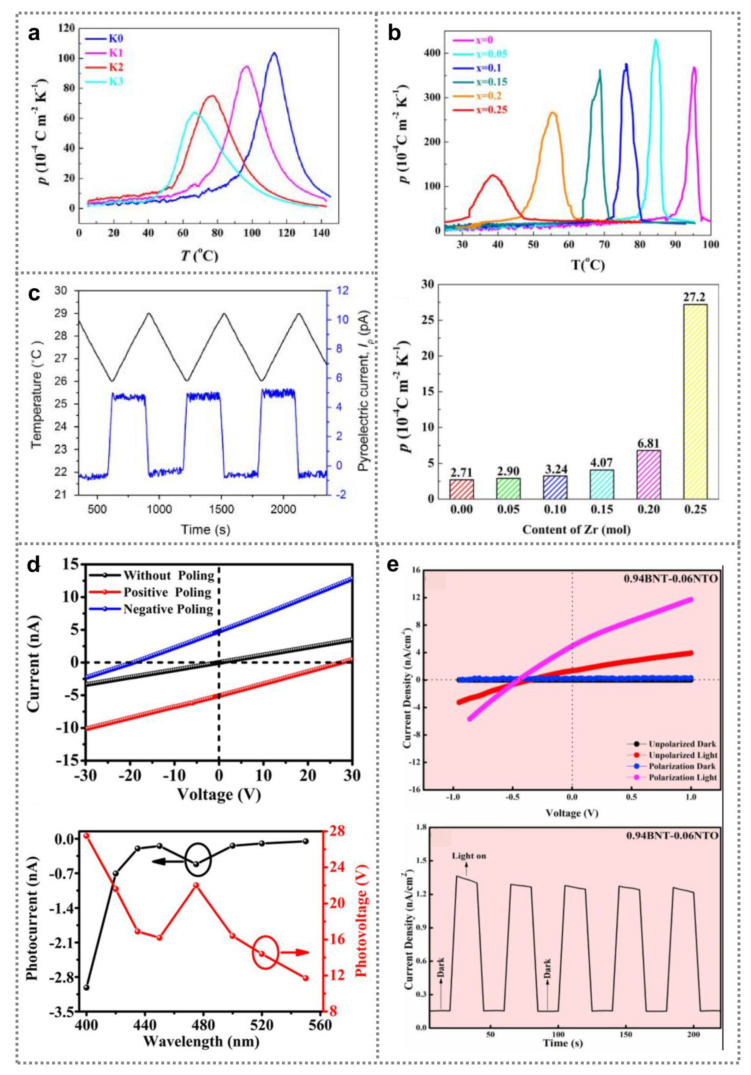
Pyroelectric and photovoltaic properties of BNT ferroelectric nanomaterials. (**a**) Temperature-dependent pyroelectric coefficients of BNT-BA-xKNN ceramic sample containing various amount of KNN (Reproduced with permission from [83], Wiley, 2019). (**b**) Pyroelectric coefficients of 0.94Bi_0.5_Na_0.5_TiO_3_-0.06BaTi_1-x_Zr_x_O_3_ ceramics with different Zr content (Reproduced with permission from [84], Elsevier, 2019). (**c**) Time-dependent temperature variation and pyroelectric current of BNT-P(VDF-TrFE) nanocomposite membranes (Reproduced with permission from [93], Elsevier, 2015). (**d**) Photovoltaic property of pure BNT ceramics (Reproduced with permission from [102], Wiley, 2020). (**e**) Current density–Voltage curves and time-dependent current density of polarized/unpolarized 0.94BNT-0.06NTO ceramics-based photovoltaic devices under different irradiation conditions (Reproduced with permission from [103], Elsevier, 2020).

The average polarization strength of ferroelectric materials can be changed with temperature, leading to pyroelectric signals in external circuit. Pyroelectric effect provides an effective approach for low-grade thermal energy harvest [77,78,79]. Pyroelectric coefficients reflect the ability of materials to produce electricity through temperature change. To efficiently collect low-grade heat, large pyroelectric coefficient is required at a lower temperature [80,81,82]. So far, a variety of research have been focused on promotion of BNT ferroelectric nanomaterials’ pyroelectricity, and remarkable progresses have been achieved. Figure 5a shows temperature-dependent pyroelectric coefficient of (1 − x)(0.98Bi_0.5_Na_0.5_TiO_3_-0.02BiAlO_3_)-x(Na_0.5_K_0.5_)NbO_3_ (BNT-BA-xKNN) ceramic samples [83], showing that the peak value of pyroelectric coefficient can be obtained at a lower temperature by increasing KNN content. Since BNT lattice’s long-range translational symmetry can be broken by KNN, ferroelectric–antiferroelectric phase transition temperature is reduced with increasing KNN content, resulting in the downshift of pyroelectric coefficient peaks. The room-temperature BNT-BA-0.02KNN ceramic samples’ pyroelectric coefficient can be as high as 80.4 μC m^−2^ K, that is two times larger than that of BNT-BA ceramic samples. Figure 5b shows the pyroelectricity of 0.94Bi_0.5_Na_0.5_TiO_3_-0.06BaTi_1-x_Zr_x_O_3_ ceramics. By adjusting Zr content, lower ferroelectric–antiferroelectric phase transition temperature was realized, leading to a large room-temperature pyroelectric coefficient (2.72 mC m^−2^ K) at x of 0.25 [84]. In addition to material composition, the pyroelectricity of BNT ferroelectric nanomaterials has exhibited strong dependence on fabrication process. For instance, by optimizing sintering temperature to 1180 °C, pyroelectric coefficient of 0.88Na_0.5_Bi_0.5_TiO_0.5_-0.084K_0.5_Bi_0.5_TiO_3_-0.036BaTiO_3_ ceramic samples can be promoted to 366 μC m^−2^ K, which was ascribed to improved density and reduced grain boundaries [85]. Recently, pyroelectricity of flexible BNT-based composite nanomaterials has been widely studied [86,87,88,89,90,91,92]. Mandi et al. investigated the pyroelectricity of BNT-P(VDF-TrFE) nanocomposite membranes [93]. By modulating BNT volume fraction to 20%, the samples obtained the maximum pyroelectric coefficient (50 mC m^−2^ K). Dc pyroelectric current can be observed by applying a triangular temperature waveform on the nanocomposite membrane (Figure 5c).

Photovoltaic effect offers a facilitate route to harvest light energy. Since multiple driving forces for carrier separation/transport and abnormal photovoltage were demonstrated in ferroelectrics, ferroelectric photovoltaic effects have attracted considerable attention [94,95,96,97,98,99,100]. Pure BNT nanomaterials possess a relatively wide energy band gap of about 3.00 eV [101], exhibiting strong ability to absorb photons in ultraviolet region. Gong et al. observed anomalous photovoltaic effect (APV) in pure BNT nanomaterials, and photovoltage as high as 27.5 V was obtained upon irradiation (405 nm, 0.2 W cm^−2^), as shown in Figure 5d [102]. Additionally, owing to oxygen vacancies, the response spectral of pure BNT ceramics can be extended to more than 500 nm. Moreover, the response spectral of BNT materials can be broaden to visible region by fabricating solid solutions. Chen et al. demonstrated role of NiTiO_3_ (NTO) content on the (1 − x)BNT-xNTO ferroelectric ceramic samples’ band gap [103]. A narrow band gap of about 2 eV was achieved in 0.94BNT-0.06NTO samples. Figure 5e exhibits photovoltaic performance of 0.94BNT-0.06NTO ceramics-based devices under standard AM1.5 irradiation (100 mW cm^−2^), showing that stable photo-response can be generated as lamp is switched on/off. Additionally, a polarized device’s short-circuit current density (*J*_sc_) as well as open-circuit voltage (*V*_oc_) can reach 5.11 nA cm^−2^ and 0.44 V, respectively, which are much higher than that of an unpolarized device. Such phenomena indicates that the ferroelectric photovoltaic effect strongly relies on the polarization state of BNT nanomaterials.

## 4. Applications

**Figure 6:**
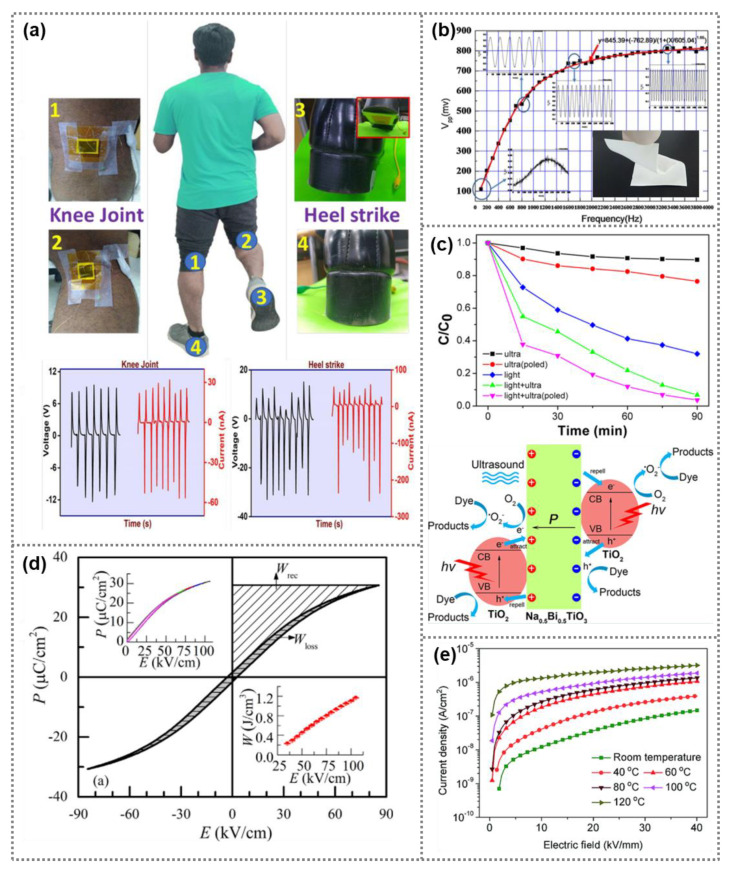
Applications of BNT ferroelectric nanomaterials. (**a**) Wearable piezoelectric nanogenerator based on BNT nanoparticles (Reproduced with permission from [105], RSC advances, 2020). (**b**) Frequency sensor based on flexible (0.78BNT-0.22ST)/PVDF composite membranes (Reproduced with permission from [47], Elsevier, 2016). (**c**) Degradation rate of RhB dye solution by using BNT@TiO_2_ heterojunction composite catalyst, and the mechanism of piezo-photocatalytic degradation of RhB by BNT@TiO_2_ nanowire with heterojunction (Reproduced with permission from [40], American Chemical Society, 2020). (**d**) Performance of (0.94 − x)Bi_0.5_Na_0.5_TiO_3_-0.06BaTiO_3_-xSrTi_0.875_Nb_0.1_O_3_ nanomaterials-based capacitors (Reproduced with permission from [107], Elsevier, 2016). (**e**) Leakage current of capacitors based on BNT/P(VDF-HFP) composite membranes (Reproduced with permission from [44], RSC advances, 2020).

Owing to the excellent ferroelectric, dielectric, pyroelectric, piezoelectric and photovoltaic properties, BNT-based nanomaterials show significant potential for numerous electronics, such as pyroelectric nanogenrator, wearable sensors, energy storage devices as well as photodetectors [22,27,52,104]. Figure 6a exhibits a wearable piezoelectric nanogenerator based on BNT nanoparticles for mechanical energy collection and motion monitoring [105]. By embedding BNT nanoparticles into polycaprolactone, piezoelectric composite membranes with outstanding flexibility were obtained. Composite membranes containing 50% BNT nanoparticles exhibited the best response to mechanical energy. The fabricated piezoelectric nanogenerator was successfully utilized to charge a capacitor, as well as monitor human’s walking and strike motions. A frequency sensor based on flexible (0.78BNT-0.22ST)/PVDF composite membranes has been constructed, as shown in Figure 6b [47]. Xu et al. synthesized BNT@TiO_2_ heterojunction composite catalyst. The BNT@TiO_2_ catalyst can degrade more than 97% of RhB dye within 1.5 h under simultaneous ultrasonic vibration and light conditions via the coupling of piezocatalytic and photocatalytic effects, and this explains the mechanism of piezo-photocatalytic degradation of RhB by the BNT@TiO_2_ composite (Figure 6c) [40]. In addition, BNT@BiOCl heterojunction composite nanomaterials have been used as photocatalyst for degradation of RhB dye solution [106]. As compared with pure BNT photocatalyst, the BNT@BiOCl heterojunction nanomaterials can more effectively separate photogenerated carriers, and restrain recombination of free electrons and holes, consequently, promoting the degradation rate of RhB dye. Additionally, capacitors on the basis of (0.94 − x)Bi_0.5_Na_0.5_TiO_3_-0.06BaTiO_3_-xSrTi_0.875_Nb_0.1_O_3_ nanomaterials were prepared, and maximum *W*_rec_ of 1.17 J cm^−3^ and *η* of 91% can be realized (Figure 6d) [107]. Moreover, 0.78(Bi_0.5_Na_0.5_)TiO_3_-0.22NaNbO_3_ ceramics-based energy storage devices have been developed. Under an electric field of 39 kV mm^−1^, large *W*_rec_ (7.02 J cm^−3^) and *η* (85%) were achieved [61]. Luo et al. constructed capacitors based on BNT/P(VDF-HFP) composite membranes [44]. The composite membrane with 2.37 vol% BNT nanofibers exhibited low room-temperature leakage current density (1.47 × 10^−7^ A cm^−2^) with high energy storage density (12.7 J cm^−3^), as shown in Figure 6e. Qiao et al. designed (1 − x)Bi_0.5_Na_0.5_TiO_3_-xSr_0.7_Sm_0.2_TiO_3_ multifunctional ceramics for simultaneous photoluminescence and energy storage applications. High *W*_rec_ (3.52 J cm^−3^) as well as high power density (220 mW cm^−3^) were obtained in 0.6Bi_0.5_Na_0.5_TiO_3_-0.4Sr_0.7_Sm_0.2_TiO_3_ ceramics [19].

## 5. Conclusions

BNT ferroelectric nanomaterials are emerging as promising functional materials for many electronics because of their large remanent polarization, excellent dielectric property, high pyroelectric and piezoelectric coefficients, and unique photovoltaic performance. By controlling structure, composition and morphology, the properties of BNT ferroelectric nanomaterials have been successfully modulated for some specific applications, such as energy harvesting, energy storage, daily lighting, photodetection and motion monitoring. Although remarkable advancements of BNT ferroelectric nanomaterials have been realized, several issues should be taken into consideration, including: (1) further reducing coercive field and increasing remanent polarization of BNT ferroelectric nanomaterials are necessary; (2) flexible BNT ferroelectric nanomaterials with more excellent piezoelectricity and pyroelectricity need to be developed for daily applications; (3) physical mechanisms of APV effect in BNT ferroelectric nanomaterials are required to be deeply revealed; and (4) approaches towards further decreasing the band-gap of BNT ferroelectric nanomaterials need to be considered. Nevertheless, with the further exploration of theory, properties and potential applications, BNT ferroelectric nanomaterials will be utilized worldwide.

## Figures and Tables

**Table 1:** Summary of morphology and synthesis methods of BNT-based nanomaterials.

Material	Nanostructures	Wavelength	Synthesis Techniques	Reference
BNT	Spheric flower	300 nm–2 μm	In situ self-assembly synthesis	[41]
BNT	Nanoplate	10–20 μm	Topochemical microcrystal conversion method	[42]
PVDF-BNT	Nanofiber	60–70 nm	Hydrothermal synthesis	[44]
BNT-BT_0.08_/CoFe_2_O_4_	Core-shell nanotube	40–45 nm	Template and sol-gel process	[48]
BNT-ST	Nanofiber	100–300 nm	Electrospinning method	[47]
BNT	Thick film	20 mm	Electrophoretic deposition	[36]
BNT-BT	Thin films	60–90 nm	Sol-gel method	[32]

**Table 2:** Summary of ferroelectric and energy storage properties of BNT nanomaterials.

Material	P_r_(μC/cm^2^)	P_max_(μC/cm^2^)	E_c_ (kV/cm)	W_rec_ (J /cm^3^)	Reference
BNT-BT-xSBT	2–32	24–43	22–46		[57]
BNT-xKN	5–41	25–47	8–52		[58]
BNT	~47	~56	~55		[59]
BNT-ST-xAN	1.6–22	32–49.5	6–23	1.5–2.5	[23]
BNT-xBNZ	18–34	60–82		24.2–50.1	[42]
BNKT	23–30	30.2–40.1	20–52	0.42–0.83	[54]
BNT-BKT-ST-xFe	14.8	71.5		11–20.34	[30]
BNT-BT	26.3	~36	27.1		[24]
BNT-xBNN	0–32	21–38	7.8–32		[45]
BNT-xBT	3–10	12.5–38			[32]
BNT-BST-xKNN		26–40		1.5–2.65	[26]
PVDF-BNT				12.7	[44]

**Table 3:** Summary of piezoelectric properties of BNT nanomaterials.

Material	Q_33_	S(%)	ε_r_	Piezoelectric Coefficient		Reference
				d_33_ (pC/N)	d_33_* (pm/V)	
BNKT-xBMT		0.15–0.4	~5300		632	[74]
BNKT-xPb		0.04–0.15	~11,000	140		[73]
BNKT-xZr		0.09–0.14	~8000	~75‘		[73]
BNT-BT				112		[24]
BNTx-BT-yKNN	0.025–0.035	0.1–0.44	~6500	181	528	[75]
BNT-BT-KN		0.1–0.2		135		[72]
BNLT-xSr		0.1–0.75	3500		~1300	[65]
BNT			885	120		[59]
BNT-xKN			380–1977			[58]
BNKT		0.12–0.35	250–550	100–160	640–720	[54]
BNT-xBNZ		0.15–0.3	1200–1500		214–428	[42]
BNT-xTi			380–502	86–98		[50]

## Data Availability

No new data were created or analyzed in this study. Data sharing is not applicable to this article.
